# Navigated Uniportal Endoscopic Decompression for Thoracic Myelopathy Secondary to Ossified Yellow Ligament: A Report of Two Cases

**DOI:** 10.7759/cureus.98572

**Published:** 2025-12-06

**Authors:** Bing Wui Ng, Ozlan Izma Muhamed Kamil

**Affiliations:** 1 Department of Orthopaedics, Prince Court Medical Centre, Kuala Lumpur, MYS

**Keywords:** endoscopic decompression, navigated endoscopy, ossified yellow ligament, thoracic myelopathy, uniportal endoscopy

## Abstract

Thoracic myelopathy secondary to ossification of the yellow ligament (OYL) is uncommon but potentially disabling. Endoscopic techniques have gained attention recently as an alternative to open decompression. We report two patients who suffered from thoracic myelopathy secondary to OYL managed using a streamlined setup of navigated uniportal endoscopic decompression utilizing only a single navigated instrument. Both patients presented with bilateral lower limb numbness and instability in walking, with a modified Japanese Orthopaedic Association (mJOA) score of 13. Both cases post-operatively demonstrated complete decompression, minimal post-operative pain, short hospital stay, and neurological recovery with improvement in mJOA scores from 13 to 17 at 10 months. These cases highlighted the advantages of the combination of endoscope and navigation, which allowed easy identification of the pathological level, the ability to visualize the trajectory needed for decompression, which ensured adequacy of removal, while intraoperative CT could verify the complete decompression.

## Introduction

Thoracic myelopathy caused by ossification of the yellow ligament (OYL) is a rare but progressive condition leading to neurological compromise. This condition occurs when progressive calcification of the ligamentum flavum narrows the spinal canal and compresses the thoracic spinal cord, leading to chronic ischemia and neural dysfunction. Surgical decompression remains the standard of care [1,2]; however, open thoracic laminectomy is associated with significant morbidity due to extensive dissection of muscles and significant postoperative pain, leading to prolonged hospital stay. Full-endoscopic techniques have demonstrated promising outcomes, with advantages including reduced blood loss, shorter hospital stays, and faster recovery compared to open surgery [3-5]. However, achieving adequate decompression or complete removal of the ossification through a limited corridor requires precise localization and control of the surgical field [6]. In recent years, navigation-assisted endoscopic techniques have been introduced to enhance surgical accuracy, especially in anatomically complex regions such as the thoracic spine [6]. The complexity of setups associated with navigation might deter surgeons from embarking on this technique. This report presents two cases of thoracic myelopathy secondary to OYL treated using a streamlined navigated uniportal endoscopic decompression technique and highlights its setup simplicity, clinical, and technical merits. To the best of our knowledge, we believe that this is the first report describing this setup and technique used in endoscopic thoracic surgery.

## Case presentation

Case 1

A 47-year-old female presented with a one-year history of gait instability and intermittent numbness in both lower limbs and trunk. She reported difficulty walking without support. Her modified Japanese Orthopaedic Association (mJOA) score was 13. MRI and CT demonstrated OYL at the T9-T11 levels.

**Figure 1:**
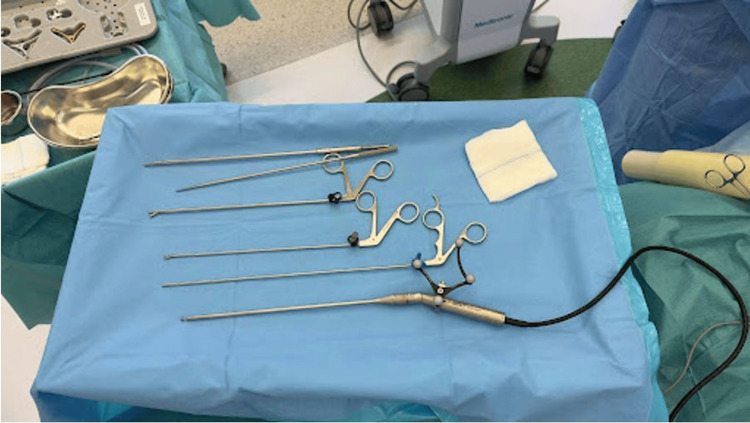
Essential instrument in performing navigated full endoscopic decompression, with the burr handpiece being the only navigated instrument.

Under general anesthesia, navigated uniportal endoscopic decompression was performed. The patient was put in the prone position on a Jackson table (Mizuho OSI, Union City, California, USA). An intraoperative preoperative CT scan was obtained using the O-arm® imaging system (Medtronic, Minneapolis, Minnesota, USA). The navigation system was registered after placing the clamp-type navigation tracker at the level of L2. The burr (NSK Primado 2 P300 handpiece; NSK Ltd., Tochigi, Japan) was the primary and only navigated instrument registered using the universal attachment (Figure 1). Real-time 3D guidance facilitated level identification, trajectory planning, and visualization of the margin of contralateral and cranio-caudal OYL.

Full endoscopic decompression was performed using the Maxmore endoscopic system (Hoogland Spine Products GmbH, Tuttlingen, Germany) with the help of a navigated burr. The posterior lamina is thinned and removed with Kerrison Rongeur. Careful removal of OYL was done with a size 1 Kerrison Rongeur and curettes. The edges of OYL could be identified with the help of direct visualization and navigation. Complete removal of OYL is checked intraoperatively by marking the margins of OYL from cranial to caudal and medial to lateral (Video 1).

**Video 1:** The navigated burr is used to determine the extent of the OYL and to facilitate its complete removal. The video demonstrates how the burr can cross to the contralateral side of the OYL under navigation guidance. OYL: ossification of the yellow ligament

**Figure 2:**
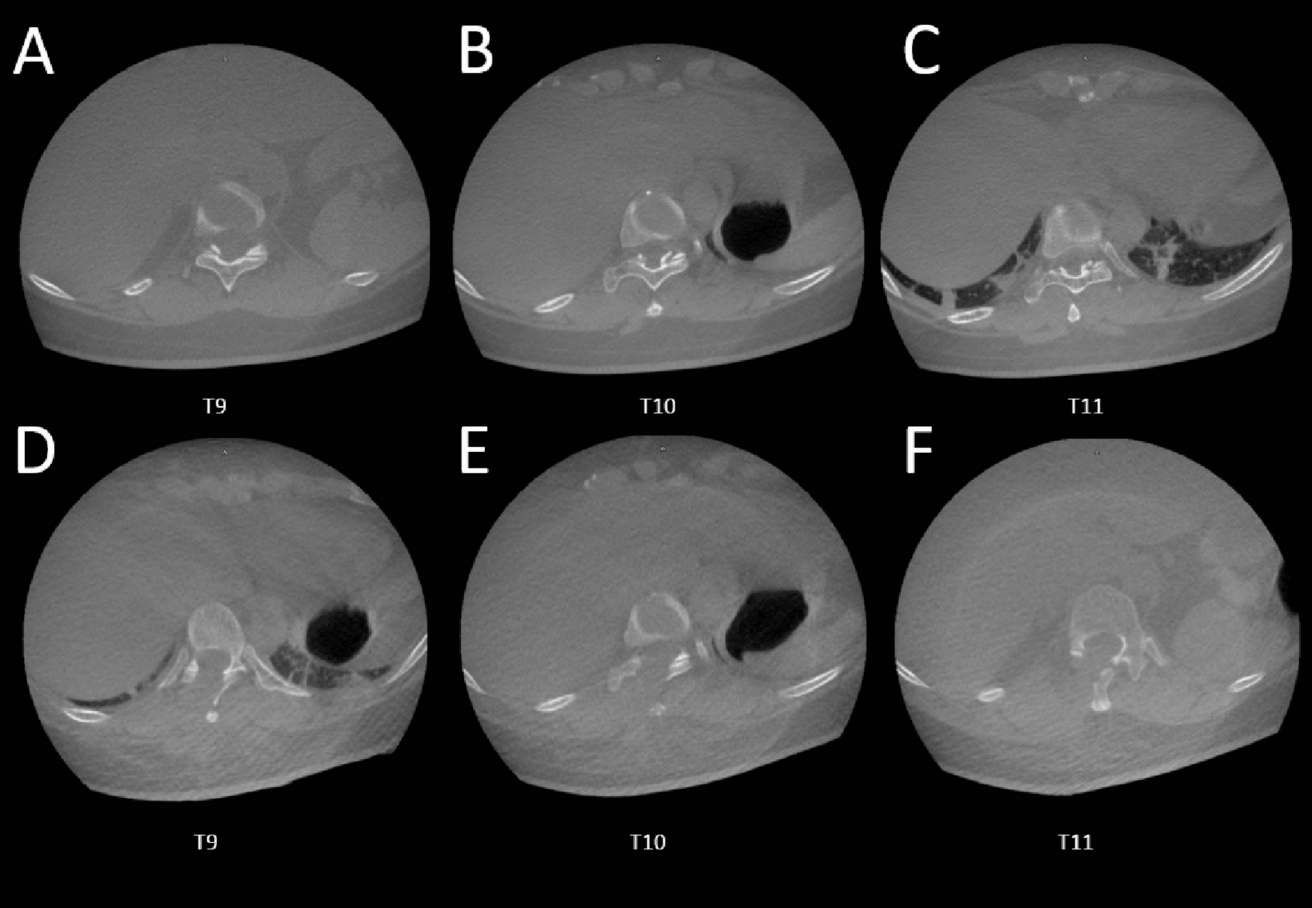
(A-C) Intraoperative CT scans demonstrating OYL at the T9, T10, and T11 levels. (D-F) Post-decompression intraoperative CT scans showing complete removal of the OYL. OYL: ossification of the yellow ligament

Intraoperative CT post-decompression confirmed complete removal of OYL (Figure 2). Postoperatively, the patient experienced mild pain at the navigation tracker site, greater than at the endoscopic incision site. The patient was discharged the next day with a Visual Analogue Scale (VAS) score of 3. At six months, her mJOA improved to 15, and she was ambulating independently. Ten months of follow-up recorded an improvement of the mJOA score to 17.

Case 2

A 53-year-old male presented with progressive gait instability. The patient could only ambulate with the aid of a walking stick and suffered from numbness in both lower limbs. Preoperative mJOA score was 12. MRI and CT scans showed OYL at T10-T11. A similar navigated endoscopic decompression was performed. However, in this case, we have used a pelvic brim navigation tracker (pin-type) to reduce post-operative discomfort.

**Figure 3:**
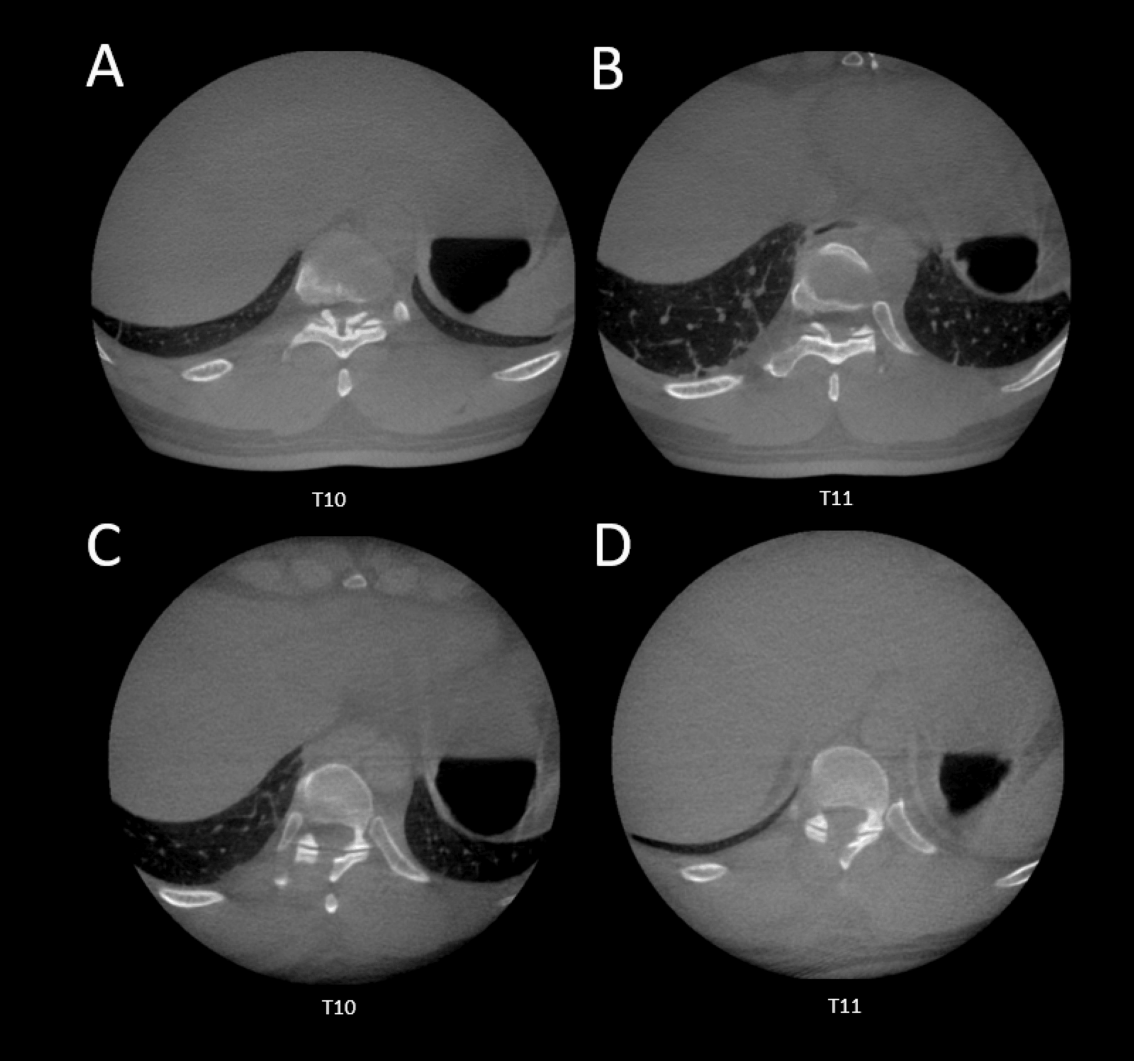
(A-B) Intraoperative CT scans demonstrating the extent of the OYL. (C-D) Postoperative CT scans showing complete removal of the OYL. OYL: ossification of the yellow ligament

Full endoscopic decompression is then performed with the technique described in Case 1. Intraoperatively, we noticed that depth variation due to respiratory motion required periodic revalidation of navigation accuracy. Post-operative CT scan prior to closing showed complete removal of OYL (Figure 3).

Postoperative pain at the pelvic tracker site was minimal. The patient was discharged the next day after the surgery with a VAS score of 1. The patient achieved significant neurological recovery with mJOA improving at six months, with reduced lower limb numbness. Follow-up during the 10th month noted that the patient improved to a mJOA score of 17.

## Discussion

Endoscopic thoracic decompression has been increasingly recognized as a viable alternative to open surgery for OYL-related myelopathy. It allows precise removal of hypertrophied ligament with minimal collateral tissue injury. However, challenges remain in achieving adequate decompression through a small working corridor [3-6]. Navigation-assisted endoscopy enhances surgical precision by providing three-dimensional (3D) visualization of the working trajectory and decompression extent [7,8]. Various types of navigation-assisted endoscopic spine surgery have been described with the use of a specific navigation pointer [9,10]. By attaching the universal navigation tracker to the burr, which is trajectory-sensitive, surgeons can benefit from real-time navigation [11]. The simplified setup described here, with navigation limited to the burr, maintains workflow efficiency and minimizes operating room complexity. However, despite the ability to understand the trajectory and location of pathology, we think that the intraoperative endoscopic view remains the main input for surgeons to perform decompression and identify end-points of the procedure. This technique offers several clinical advantages. With navigation, the operative level can be easily identified in real-time via 3D navigation images [11]. Rather than using multiple fluoroscopy shots from the neck down or waist up and placing multiple check-point needles to identify the level of disease, navigation provides a straightforward way to identify the level of disease by placing the navigated instrument on the skin before incision. This also allows the surgeon to plan the incision and trajectory, which will eventually enable him to address the contralateral decompression at a comfortable angle with the endoscope [6,12].

Besides that, the endoscopic technique has been shown to reduce tissue dissection, blood loss, and shorten the duration of hospitalization [5,13]. As described in the first case, the patient complained of more significant discomfort at the site of navigation tracker insertion rather than the endoscopic incision for the surgery. By changing to a pin-type tracker, the discomfort could be further reduced.

Furthermore, intraoperative CT verification after the surgery, before closing the wounds, is one of the best modalities to confirm adequate decompression [14]. The surgeon will be able to address any residual compression by reviewing the post-decompression CT intraoperatively. This is essentially the most beneficial step in ensuring complete decompression. Despite these benefits, there are certain technical challenges. Navigation accuracy may be affected by thoracic movement during respiration or changes in patient positioning. Extreme care and planning must be put into deciding the location and direction of the tracker, so as not to accidentally change its position during the surgery. Subtle misalignment over time requires periodic re-validation of navigation accuracy during surgery. Larger clinical series are necessary to validate the reproducibility and long-term outcomes of this technique.

## Conclusions

Navigated uniportal endoscopic decompression is a feasible and effective option for thoracic myelopathy secondary to OYL. It combines the visualization and tissue-sparing benefits of endoscopy with the precision of navigation. The simplicity in setup, with the utilization of only one navigated instrument, could streamline the surgical process. These two cases highlight the effectiveness and safety of this technique in treating thoracic myelopathy secondary to OYL. The navigated instrument enables correct identification of the level and margins of the OYL, coupled with the intraoperative CT post-decompression, further ensuring the complete removal of the OYL. Despite minor workflow and accuracy limitations, this technique enhances surgical safety and confidence, particularly by ensuring the complete removal of OYL. Further studies with larger cohorts are warranted to confirm long-term outcomes.
